# Symbolic Number Abilities Predict Later Approximate Number System Acuity in Preschool Children

**DOI:** 10.1371/journal.pone.0091839

**Published:** 2014-03-17

**Authors:** Christophe Mussolin, Julie Nys, Alain Content, Jacqueline Leybaert

**Affiliations:** Center for Research in Cognition and Neurosciences, Laboratory Cognition Language Development, Université Libre de Bruxelles, Brussels, Belgium; Birkbeck, University of London, United Kingdom

## Abstract

An ongoing debate in research on numerical cognition concerns the extent to which the approximate number system and symbolic number knowledge influence each other during development. The current study aims at establishing the direction of the developmental association between these two kinds of abilities at an early age. Fifty-seven children of 3–4 years performed two assessments at 7 months interval. In each assessment, children's precision in discriminating numerosities as well as their capacity to manipulate number words and Arabic digits was measured. By comparing relationships between pairs of measures across the two time points, we were able to assess the predictive direction of the link. Our data indicate that both cardinality proficiency and symbolic number knowledge predict later accuracy in numerosity comparison whereas the reverse links are not significant. The present findings are the first to provide longitudinal evidence that the early acquisition of symbolic numbers is an important precursor in the developmental refinement of the approximate number representation system.

## Introduction

Adults, young children, and infants are able to detect differences in numerosity represented by visual elements, sounds, or actions well before the acquisition of language [1]–[3]. These abilities are assumed to depend on a specific representational system, known as the approximate number system (ANS), which takes the form of a noisy representation of number magnitude obeying Weber-Fechner's law [4]. Even newborn infants show the ratio-dependent numerosity performance that is characteristic of older children and adults [5]. Furthermore, ANS acuity changes dramatically from infancy to adulthood, suggesting a progressive refinement of internal number representation over life span. Six-month-old infants discriminate visual arrays or sequences of tones above chance when numerosities are in a 1∶2 ratio but not when a 2∶3 ratio is used [6], [7]. Nine-month-olds succeed with the 2∶3 ratios with which 6-month-olds fail [7]. Later on, children achieve above chance comparison performance with numerical ratios that increase monotonically from 2∶3 to 6∶7 between the age of 3 and 6 [8]. The refinement continues to reach a 9∶10 ratio in adults [8], [9].

Beyond those preverbal abilities, human beings are able to represent the numerosity of collections of individual objects with unit precision. Children first acquire symbolic capacities through verbal counting, which implies mastering the sequence of number words, and understanding the inherent principles of the counting procedure [10]. This is a long-lasting process that starts around the age of 2 and goes on up to about the age of 6. At around 4 years of age, children also start to recognize and manipulate Arabic digits. They learn that each of these visual symbols refers to an exact quantity by mapping them onto the corresponding numerosities. Both behavioural [11] and neuroimaging [12] data indicate that, from the age of 5–6, children do access the magnitude of Arabic digits as indexed by the symbolic distance effect. In both children and adults, comparing two digits separated by a small numerical distance such as 1 (e.g., 6 vs. 7) is slower and more error-prone than digits separated by a larger numerical distance such as 3 (e.g., 6 vs. 9) [13]. The size of this effect decreases with age, possibly reflecting increasing precision in the comparison between numbers during development [11], [14].

Different theories have been proposed to explain how children acquire the meaning of symbolic numerals. It is widely assumed that this learning is done by mapping number words or Arabic digits onto the pre-existing approximate number representation. As a direct consequence, the ANS would play a crucial role in the early foundation of symbolic number knowledge [10], [15], [16]. Based on another view, Carey [17] states that the child ascertains the meaning of “two” from the interplay of acquisition of natural language quantifiers with the visual attention system of parallel object individuation, whereas he/she comes to know the meaning of “five” through the bootstrapping process - i.e., that “five” means “one more than four, which is one more than three.” - by integrating representations of natural language quantifiers with the external serial ordered count list. Therefore, children need not map large numerals onto large analog magnitudes to acquire their meaning. It is only after the acquisition of the cardinality principle that children would start to map number words to the ANS [18].

Up to now, developmental data on the relationships between numerosity acuity and performance in tasks involving number words or Arabic digits are unable to disentangle the above theories. A first line of research examining the link between accuracy in numerosity comparison and counting skills led to controversial results. On the one hand, some studies reported that preschool children who possess some understanding of the cardinality principle show higher precision in discriminating among sets of visual objects varying in numerical ratios than children who had no or only little cardinality knowledge [19]–[21]. On the other hand, no correlation was found between the highest number of objects correctly counted and performance in a task requiring to approximately represent the outcomes of subtraction operations on large sets [22], nor between accuracy in discriminating numerosities and performance in the “How many?” task [23].

Halberda and his colleagues [24] initiated a second line of research by showing that the individual differences in ANS acuity at 14 years were related to math achievement scores from kindergarten to sixth grade, even after control for general cognitive factors. Since this work, further experiments have also found such a link in preschool children [25] and school-aged children [26]. It is however worth noting that others have failed to establish a relationship between non-symbolic number processing and arithmetic achievement. Several studies with school-aged children [14], [27], [28] showed that arithmetic scores were negatively correlated with the distance effect for Arabic digits but not with the distance effect observed for the corresponding non-symbolic quantities. In adults, current results are also mixed since no consistent relationship between ANS acuity and math was found [26], [29]. Several studies reported a significant correlation between performance in numerosity comparison and mental arithmetic in adults [30], [31], but this link could be mediated by basic symbolic number knowledge such as the ability to order Arabic digits [32].

None of the available developmental studies provided evidence indicating the direction of the influence. Mazzocco, Feigenson, and Halberda [33] have shown that the accuracy in numerosity comparison measured in children of 3-6 years could predict their school mathematics performance two years later. The authors take those findings as evidence favouring the view that symbolic number abilities depend on ANS acuity. However, we and others have reported extremely large variability in knowledge about symbolic numbers at preschool [18], [21], [34]. Between 3 and 5 years, the degree of mastery of the meaning of number words differs largely across children. While some of them grasp the concept of cardinality (i.e., Cardinality Principle knowers), others understand only what “one”, “two”, or “three” represents (i.e., subset knowers). Mazzocco et al. [33] did not take into account children's knowledge about symbolic numbers like cardinality proficiency during the first assessment. Therefore, the possibility that these initial differences, rather than variability in ANS acuity, would explain the later scores on maths could not be rejected. In an attempt to fill this gap, Libertus and colleagues [35] tested the relationship between ANS acuity and math ability in a large sample of 4-year-olds while taking individual differences on a standardized math battery into account. They found that accuracy in numerosity comparison contributed uniquely to the relationship with math ability 6 months later, even when controlling for the initial math score. However, the percentage of explained variance was very weak and the question of the reverse relationship has not been investigated.

In the present study, three theoretical accounts concerning the association between ANS acuity and symbolic number abilities in young children are assessed: First, ANS acuity enhances or accelerates the acquisition of symbolic number knowledge; second, the manipulation of symbolic numerals has an impact on the precision of ANS; third, both relationships operate simultaneously, so that ANS acuity and symbolic number abilities have a reciprocal influence. These hypotheses were tested by asking 57 children of 3–4 years to participate in two testing assessments administered at an interval of seven months. The first assessment contained a numerosity comparison task as an index of ANS acuity, different tests assessing knowledge about number words and Arabic digits, and general cognitive factors like verbal and visuospatial short-term memory spans, non-verbal intelligence, and language comprehension. The second assessment comprised the numerosity comparison and symbolic number tasks only.

Cross-lagged correlations were used to test the relationships between ANS acuity and symbolic number abilities. This method involves contrasting the correlations obtained between two variables [36]–[38] such as numerosity comparison and symbolic number knowledge across two time points in a longitudinal study. By comparing the strength of associations, such as the correlation between performance in numerosity comparison at time point 1 with scores on symbolic number tasks at time point 2 and the correlation between scores on symbolic number tasks at time point 1 with performance in numerosity comparison at time point 2, we will be able to assess the predictive direction of the relationship. According to the view that the precision of the ANS leads to faster acquisition of symbolic number knowledge, we should expect a larger correlation between performance in numerosity comparison at time point 1 with scores on a symbolic number tasks at time point 2 than in the reverse correlation between scores on symbolic number tasks at time point 1 with performance in numerosity comparison at time point 2. By contrast, if the correlation between numerosity comparison at time point 1 and symbolic number measures at time point 2 is significantly lower than the correlation between symbolic number measures at time point 1 and numerosity comparison at time point 2, the hypothesis that ANS acuity exerts a stronger influence on later symbolic number abilities than vice versa might be rejected. Finally, if ANS acuity and symbolic number abilities influence each other, we should obtain similar correlations between both measures across the two time points.

## Method

### Ethics statement

The research procedures described below were completed in accordance with approval from the Institutional Review Board at the Brussels University. The research was conducted in Belgium. All protocols have been conducted according to the principles expressed in the Declaration of Helsinki. All legal guardians of the children gave informed written consent prior to the experiment.

### Participants

Fifty-seven preschool children (27 girls and 30 boys, mean age  =  4 years, range  =  3 years–4 years 9 months) were recruited from a large sample who had participated in a cross-sectional study testing the link between ANS acuity and symbolic number knowledge [34]. For the present longitudinal study, only 3- (5 girls and 11 boys, mean age  =  3 years 5 months, range  =  3 year – 3 years 10 months) and 4-year-old children (22 girls and 19 boys, mean age  =  4 years 3 months, range  =  3 years 8 months–4 years 9 months) were invited to return for a follow-up testing seven months later and took part in the second assessment. Twenty-three additional children (twenty 3-year-olds and three 4-year-olds) participated in the study but were excluded because their performance in numerosity comparison was below chance (19) or because they had not attended the second session (3).

#### Tasks and procedure

At both testing occasions, children were seen individually in a quiet room in their school. The first assessment included two sessions of approximately 20 min with a week in-between. Children had to perform the different numerical and non-numerical tasks (described hereafter). The second assessment included one session in which children performed only the numerical tasks. In each assessment, the order of the tasks was counterbalanced across participants (described elsewhere in greater details [34]).

#### Numerosity comparison

We assessed children's performance in a computer-based numerosity comparison task as a measure of ANS acuity. Children sat at a table next to the experimenter in front of the laptop. Trials consisted of two successive sets of train wagons presented on each side of a 17-inch colour screen, and children had to select the set containing more wagons. Each wagon set was associated with a cartoon character and children were asked to indicate which of them “wins” by selecting the correct one. The ratio between the two sets varied from 1∶2 to 7∶8. Given that small numerosities involve different quantification processes than large ones [39], only numerosities above three (except for ratio 1∶2) were presented (see Table 1). The trials were controlled for area and external perimeter to ensure that responses were based on the number of wagons and not on non-numerical perceptual variables. In each pair, both collections of wagons were displayed within a similar virtual rectangle, keeping the external perimeter constant. The total surface area of the wagons, which corresponded to the sum of the area occupied by each wagon, was equated in each pair by reducing the length of the items in the collection with more wagons, while height was kept constant for all wagons. To avoid the more numerous collection being also the one with the smaller elements, wagons of different lengths were used and the length of the smallest (as much as possible) and largest wagons was the same in both arrays to be compared. Furthermore, the external perimeter of both collections was identical within each pair.

**Table 1:** The 14 pairs of numerosities used across the different ratios.

Ratio	Set size	
	1	2
1:2	3 vs. 6	4 vs. 8
2:3	4 vs. 6	6 vs. 9
3:4	6 vs. 8	9 vs. 12
4:5	4 vs. 5	12 vs. 15
5:6	5 vs. 6	15 vs. 18
6:7	6 vs. 7	18 vs. 21
7:8	7 vs. 8	21 vs. 24

The task was run on a laptop using E-Prime 1.2 software [40]. Each trial corresponded to the same sequence of events. First, a fixation point was shown for 200 ms, followed by the two arrays successively presented on each side of the screen for a variable duration depending on the ratio, with an inter-stimuli interval of 200 ms. As the size of numerosities globally increased with ratio, display time was adjusted by ratio (i.e., 900 ms for 1∶2 ratio, 1000 ms for 2∶3 ratio, 1400 ms for 3∶4 ratio, 1800 ms for 4∶5 ratio, 2200 ms for 5∶6 ratio, 2600 ms for 6∶7 ratio, and 3000 ms for 7∶8 ratio), with a duration chosen through pilot testing to be long enough to allow participants to view all elements in each array but short enough to prevent serial counting. The wagon pictures were displayed in black on a grey background. Then, a screen with the two cartoon characters at the place of each corresponding array appeared for 3000 ms during which the child could produce his/her response, indicating which cartoon character was associated with the highest number of train wagons. Children were asked to respond orally or to point to the character on the screen, and the experimenter pressed the corresponding left- or right-hand key on a two-button response box. Reaction times (RTs) were recorded from the onset of the second array until the experimenter's response. A feedback consisting of a positive tone was produced irrespective of the correctness of the response. Finally, an empty screen was used as inter-trial interval for 2000 ms. In all tasks, the position of the first array and the position of the correct response were counterbalanced across trials. Participants completed a practice block that consisted of four trials with 1∶2 ratio and were then given successive blocks (one per ratio) of 8 stimuli. To measure children's ability to discriminate numerosities, we used an increasing staircase procedure. As the precision in numerosity comparison is characterized by a ratio-limit performance that varies largely across participants [24], when accuracy falls below chance level for a specific ratio, no better performance is expected for more difficult ratios. Thus, participants first performed trials corresponding to three easiest ratios (1∶2, 2∶3, 3∶4), exactly as in previous studies on young children [20], [21]. Then, they were successively presented with the next finer ratios (4∶5, 5∶6, 6∶7, 7∶8), with one ratio per block, until accuracy fell below 75% (6 out of 8 correct responses) within a block. This technique allowed us to precisely assess the level of acuity reached by each child as the highest ratio she/he could successfully discriminate above chance (*χ* ^2^(1)  = 4.5, *p*<.05) with a restricted number of stimuli. In line with past research on young children [21], [33], the correct response rate (i.e., computed on the number of trials received by each child) is taken as the main measure of individual ANS acuity throughout the present paper.

Participants also performed a counting task in which they were presented with similar numerosities and had to count the number of wagons aloud as fast as possible and to produce an oral response. Measuring the latencies in this task allowed us to exclude the possibility that children used counting strategies in the numerosity comparison task.

#### Symbolic number knowledge

Three measures of symbolic number abilities were taken at each time point. First, we assessed children's *counting list* by asking them to count up to sixty. The highest number word that each child could produce in the correct sequence was taken as a measure of the counting range. The second measure concerned the cardinality proficiency score using the *give-a-number task* [16]. Each child was presented with a set of 10 small plastic animals (dog, cow, sheep or pig, flamingo, zebra) placed in a small bowl and was asked to take *x* dogs out of the bowl and put it on the table where *x* varied from 1 to 5, in a pseudo-randomized order until all numerosities were tested. Additional trials including seven animals were presented to 4-year-olds. Children performed the give-a-number task with each of the three animal sets in different orders, giving a total of fifteen or eighteen trials. Finally, children received a battery that provides a measure of symbolic number knowledge (described elsewhere in greater details [34]). This *symbolic battery* includes several tests that assess the ability to manipulate the number word sequence [41]; to recognize, name and compare Arabic digits; to automatically access the cardinality represented either by canonical dot patterns or by the number raised on a hand; to identify number words (adapted from a subtest of TEDI-MATH battery [42]); and to estimate the plausibility of numerical estimations in real context (adapted from a subtest of ZAREKI battery [43]). Altogether, a maximum score of 63 could be reached. To ensure that all subtests of the symbolic battery refer to a common mechanism, we conducted a factor analysis. Only one significant component was extracted by the analysis which accounted for 51% of variance. The component matrix indicated that all symbolic number scores had stronger saturations on this component (from .64 to .80), suggesting that they all are characterized by a common mechanism of symbolic number processing. The symbolic battery had a good reliability and internal consistency, indexed by a Cronbach's alpha of .68, given the heterogeneity of the tasks.

#### General cognitive factors

Beyond numerical tasks, general cognitive factors were assessed. Intellectual abilities were evaluated using Raven's Coloured Progressive Matrices [44], which are extensively used as a test of non-verbal intelligence with children. In the verbal short-term memory task, children were presented with increasingly longer series of pseudowords from a standardized battery of verbal language assessment in young children [45] and were asked to repeat them in the actual presentation order. The Corsi block-tapping test [46] provided a measure of visuospatial short-term memory, in which children were asked to reproduce the same sequence of block tapping as shown by the examiner. In both tasks, the span is reflected by the highest level passed by the child. Finally, language production was assessed by a subtest of the NEEL (Les Nouvelles Epreuves pour l′Examen du Langage [47]), a French standardized battery that is used to measure naming performance from the age of 3.

## Results

### Longitudinal analyses

#### Numerosity comparison

Table 2 reports the changes in performance across time for each numerical task, as well as simple paired *t*-tests for the improvements. Regarding the numerosity comparison task, the mean overall accuracy across all ratios performed by each child improved from 69% to 80% across the two time points. In both testing sessions, performance decreased as the ratio between the two numerosities increased (from 73% to 53% at time point 1; from 88% to 64% at time point 2), providing evidence of approximate representation. A repeated-measures analysis of variance (ANOVA) with ratio (1∶2, 2∶3, 3∶4) and time (time point 1 vs. time point 2) confirmed that both effects were highly significant (ratio: *F*(2, 110)  = 59.58, partial η^2^ = .52, *p*<.001; time: *F*(1, 55)  = 27.74, partial η^2^ = .33, *p*<.001). Children's performance decreased as the ratio between numerosities increased. Post-hoc *t*-tests revealed that correct response rates were lower at the 3∶4 ratio (60%) than at the 1∶2 and 2∶3 ratios (83% and 81% respectively, *p*s<.001) that did not differ from each other (*p* = .49). Accuracy in the three easiest ratios improved across the two time points (from 66% to 83%). As indicated by the Ratio × Time interaction (*F*(2, 110)  = 7.84, partial η^2^ = .12, *p* = .001), the improvement was stronger for ratio 1∶2 (*F*(1, 55)  = 35.15, partial η^2^ = .39, *p*<.001; from 75% to 91%) and 3∶4 (*F*(1, 55)  = 19.32, partial η^2^ = .26, *p*<.001; from 48% to 73%) than for ratio 2∶3 (*F*(1, 55)  = 9.75, partial η^2^ = .15, *p* = .003; from 77% to 82%).

**Table 2:** Performance in each task at time points 1 and 2.

	Time point 1	Time point 2	Paired *t*-test
	Mean (*SD*)	Mean (*SD*)	*df t*
Accuracy in numerosity comparison (%)	69 (13.5)	80 (10.4)	56 -7.51***
Weber fraction	.42 (.21)	.26 (.17)	49 5.10***
Give-a-number task (/15 or/18)	13 (4.6)	14.5 (4.2)	56 -3.94***
Counting list (highest number word)	13 (6)	21 (12)	55 -6.57***
Symbolic number battery (/63)	30 (18.4)	38.8 (17.4)	55 -6.86***
Intellectual abilities	13 (3.6)	-	-
Verbal short-term memory span	3.45 (.7)	-	-
Visuospatial short-term memory span	2.4 (.8)	-	-
Language abilities	38.6 (12.9)	-	-

*Note*. The Weber fractions were computed only for 52 and 53 children in time points 1 and 2 respectively due to low performance exhibited by other children.

***p<.001.

Several reasons lead to exclude the possibility that children used subitizing (as the ability to enumerate a small group of four or fewer objects fast and accurately without counting [48]) or counting to apprehend the number of wagons. First, all numerosities were above three except in one pair with the 1∶2 ratio, limiting the opportunity to use subitizing. Second, participants' counting skills were not sufficiently efficient to be used in the numerosity comparison task. Indeed, the majority of 3- and 4-year-olds needed to point to the wagon pictures when they were required to count numerosities out loud. Third, only 14 and 18 of the 57 children understood the cardinality principle at time points 1 and 2 respectively, whereas the others knew the exact meaning of only a few number words. Fourth, the RTs in the numerosity comparison task were at least two times shorter than the latencies in the counting task for all numerosities (*p*s <.001). Finally, if children used counting strategy to select the larger numerosities, one could expect a relationship between RTs and correct response rates in the comparison task at least for trials including the smallest numerosities (i.e., the three easiest ratios). However, there was no significant correlation between latencies and accuracy in either 3-year-olds (time point 1: *r*(16)  = −.23, *p* = .38; time point 2: *r*(16)  = −.37, *p* = .15) or 4-year-olds (time point 1: *r*(41)  = .005, *p* = .98; time point 2: *r*(41)  = .09, *p* = .57).

To obtain another measure of children's sensitivity to numerosity comparison, we also computed the internal Weber fraction (*w*) on accuracy across the different ratios. The Weber fraction reflects the smaller ratio needed to reliably detect a difference between two stimuli. Thus lower values of *w* index finer ANS acuity. Based on past research [24], the value of *w* was estimated for each child from the proportion of correct responses by fitting a normal cumulative probability distribution modelling the difference between the two numerosities. We used an iterative algorithm performing non-linear least-squares fit on the proportion of correct responses for all available ratios (fitting parameters are described elsewhere [49]). In line with the estimates obtained in 3- and 4-year-olds in earlier studies [8], the mean value of participants' *w* parameters decreased from .42 at time point 1 to .26 at time point 2. Note that 5 children at time point 1 and 4 children at time point 2 obtained a Weber fraction above 1, corresponding to a lower acuity than that observed in 6-month-olds during numerosity habituation [6]. These children were thus excluded from subsequent correlations involving the *w* parameter.

#### Symbolic number measures

Children's performance also improved in the three symbolic measures across time. Of the 57 participants, forty were able to count above ten at the time point 1 and only three of them produced a sequence above twenty. Seven months later, 48 children were able to count above ten and twenty-one counted up to twenty or above. The score on the give-a-number task also increased from 13 to 14.5. Based on the typical classification of children [18], [21], children are classified as pre-knowers when they have not yet assigned an exact meaning to any of the numerals, and as subset knowers when they know the exact meanings for only a subset of those numerals. Cardinality levels ranged from pre- to seven-knowers at the first assessment while the majority of children were distributed in the highest levels of proficiency at the second assessment (see Table 3). Compared with their initial performance, two 3-year-olds and four 4-year-olds reached the highest level of cardinality seven months later although the distribution of participants according to cardinality was not significantly different at the two time points (*χ* ^2^(6)  = 7.42, *p*>.10). The score on the symbolic battery also improved with age from 30 to almost 39.

**Table 3:** Number of children in the different knower-levels at the two time points by age group.

	Knower-level	Time point 1	Time point 2
3-year-olds	Pre-knowers	1	-
	One-knowers	5	2
	Two-knowers	5	7
	Three-knowers	1	1
	Four-knowers	2	2
	Five-knowers	2	4
4-year-olds	Pre-knowers	1	-
	One-knowers	-	-
	Two-knowers	8	2
	Three-knowers	2	1
	Four-knowers	7	4
	Five-knowers	9	16
	Seven-knowers	14	18

### Correlations at each time point

#### Simple correlations

Table 4 reports simple and age-controlled correlations for every pair of measures at each time point. The majority of the correlations were highly significant even when controlled for age. Of particular concern here is the link between measures of ANS acuity indexed by either accuracy or the *w* parameter and performance in the give-a-number task, counting list, and symbolic battery. At both time points, significant correlations were found between each combination of non-symbolic and symbolic number measures, except for the counting list, which showed weaker correlations or sometimes no link with ANS acuity. These findings indicate that 3- and 4- year-old children who had a more precise (a less imprecise) approximate number representation tended to perform better on the different symbolic number measures.

**Table 4:** Correlations between symbolic number measures and sensitivity to numerosities at the two time points.

Factors	1	2	3	4	5	6	7	8	9	10
1. Numerosity comparison, Time 1		−.62***	.38**	.27*	.41**	.43**	−.35*	.26†	.21	.31*
2. Weber fraction^a^, Time 1	−.68***		−.34*	−.15	−.10	−.17	.23	−.15	−.07	−.10
3. Give-a-number score, Time 1	.62***	−.48***		.45***	.50***	.43**	−.20	.60***	.26†	.45**
4. Counting list, Time 1	.51***	−.31*	.65***		.47***	.27*	−.03	.50***	.52***	.48***
5. Symbolic battery score, Time 1	.67***	−.33*	.75***	.68***		.52***	−.48***	.68***	.40**	.71***
6. Numerosity comparison, Time 2	.61***	−.32*	.62***	.48***	.68***		−.81***	.67***	.21	.58***
7. Weber fraction^a^, Time 2	−.55***	.35*	−.49***	−.31*	−.69***	−.85***		−.48***	−.07	−.47***
8. Give-a-number score, Time 2	.58***	−.35*	.80***	.69***	.86***	.76***	−.68***		.41**	.81***
9. Counting list, Time 2	.40**	−.19	.47***	.63***	.57***	.39**	−.28*	.59***		.42**
10. Symbolic battery score, Time 2	.59***	−.32*	.71***	.67***	.86***	.71***	−.67***	.91***	.57***	

*Note.* The area below the diagonal presents bilateral Pearson correlation coefficients, and the area above the diagonal reports partial correlations controlling for age.

a The Weber fractions were computed only for 52 and 53 children in time points 1 and 2 respectively due to low performance for other children.

† *p* = .05, *p<.05, **p<.01, ***p<.001.

#### Partial correlations

To check for the possibility that the link between ANS acuity and symbolic number abilities was mediated by general cognitive factors, we calculated partial correlations between each pair of non-symbolic and symbolic numerical measures, with age, non-verbal intelligence, visuospatial and verbal short-term memory as well as naming performance controlled for.

Note that one child did not understand the verbal short-term memory task and five children did not understand the visuospatial short-term memory task; they were thus excluded from the correlation analyses. At both time points, children showed positive correlations between accuracy in numerosity comparison and the give-a-number task score (time point 1: *r*(45)  = .35, *p* = .02; time point 2: *r*(45)  = .62, *p*<.001) or the symbolic battery score (time point 1: *r*(45)  = .37, *p* = .01; time point 2: *r*(45)  = .53, *p*<.001). Only the correlation with the counting list failed to reach significance when general cognitive factors were controlled for (time point 1: *r*(45)  = .17, *p* = .24; time point 2: *r*(45)  = .20, *p* = .17). Similar results were obtained when considering the *w* parameter as an index of ANS acuity. After partialling out the effects of age, non-verbal intelligence, and short-term memory, significant negative correlations were observed with the give-a-number score at both time points (time point 1: *r*(42)  = −.32, *p* = .03; time point 2: *r*(43)  = −.36, *p* = .02), as well as with the symbolic battery score at time point 2 only (time point 1: *r*(42)  = .02, *p* = .86; time point 2: *r*(42)  = −.38, *p* = .01). By contrast, the *w* parameter did not correlate with the counting list (time point 1: *r*(42)  = −.06, *p* = .70; time point 2: *r*(42)  = .01, *p* = .97).

### Correlations across time points

Although the above correlations support the notion of a specific relation between ANS acuity and symbolic number knowledge, they provide no indication on the predictive direction of the relation. So far, the results are in line with the view that ANS acuity influences the development of symbolic number abilities, but they are also compatible with the alternative account that the acquisition of numeral knowledge affects the precision of ANS. Regarding the simple correlations, the degree of association between each of the symbolic number measures (i.e., give-a-number task, symbolic battery, counting list) at time point 1 and accuracy in numerosity comparison at time point 2 was higher than the reverse associations. A similar predictive direction emerged when the Weber fraction (*w*) was taken as another index of ANS acuity.

**Figure 1:**
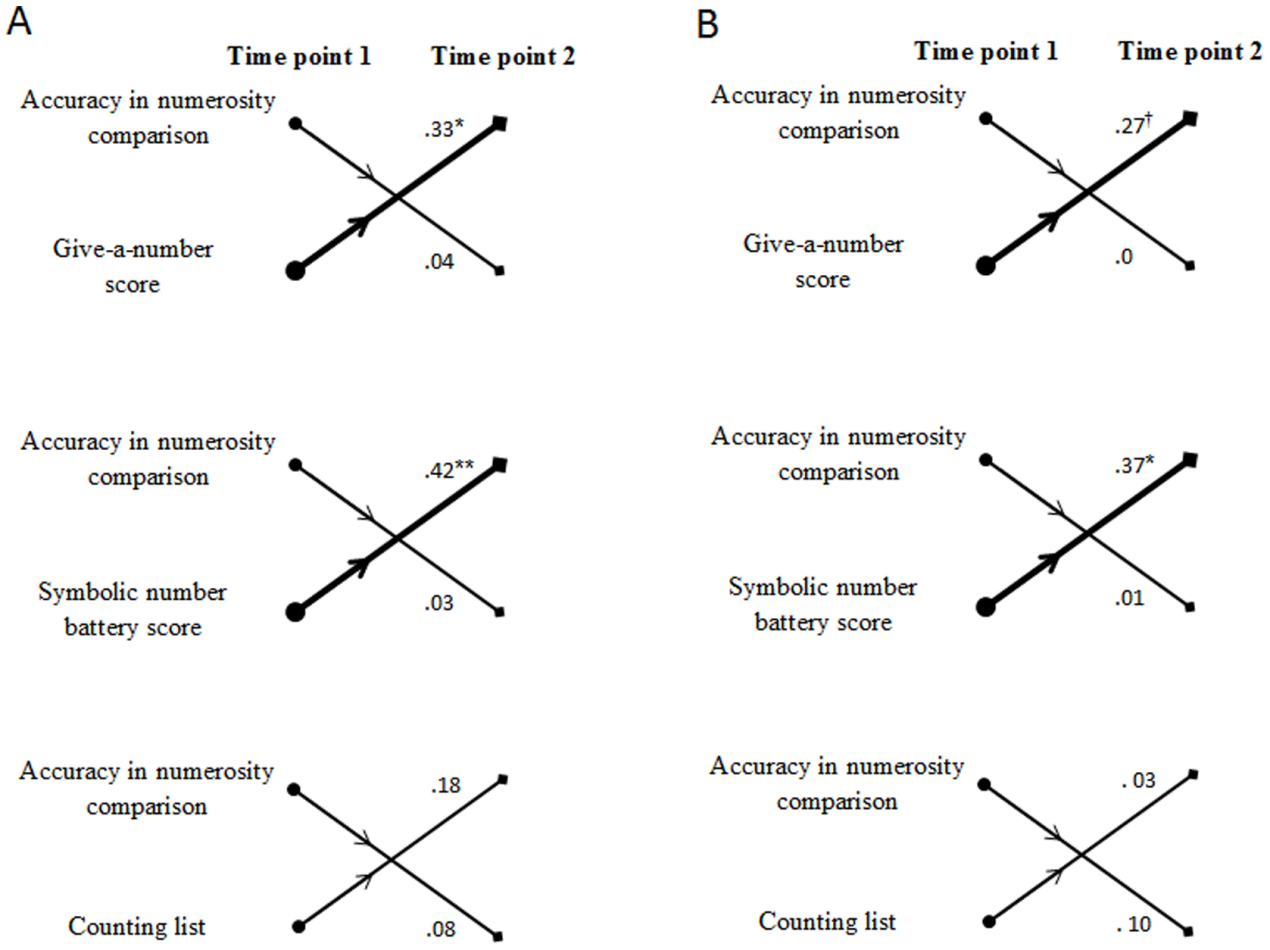
Cross-lagged correlations between each symbolic number measure and accuracy in numerosity comparison after controlling for age and the contribution of the autoregressor (Panel A) and general cognitive factors (Panel B). Significant partial correlations are marked by *p = .01, **p = .001; a tendency by a ^†^.05<*p*<.10. Lines shown in bold denote the one of the two cross-lagged correlations that is significantly greater.



To disentangle the different theoretical accounts, cross-lagged correlations between the three symbolic number measures and accuracy in numerosity comparison were examined across the two time points (see Figure 1). Then, we analyzed the reciprocal relationships between the three symbolic number measures and the other index of ANS acuity, that is, the internal Weber fraction. To compare the strength of the two cross-lagged correlations, we used the test established by Williams [50] and then validated by Steiger [51]. To do that, we calculated the difference between the two non-independent correlations by taking into account the degree of association of the two predictors, as follows:

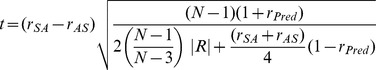
where
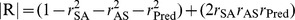



*r*_SA_  =  correlation between symbolic number skills at time point 1 and ANS acuity at time point 2

*r*_AS_  =  correlation between ANS acuity at time point 1 and symbolic number skills at time point 2

*r*_Pred_  =  correlation between ANS acuity at time point 1 and symbolic number skills at time point 1

#### Symbolic number measures and accuracy in numerosity comparison

**Figure 2:**
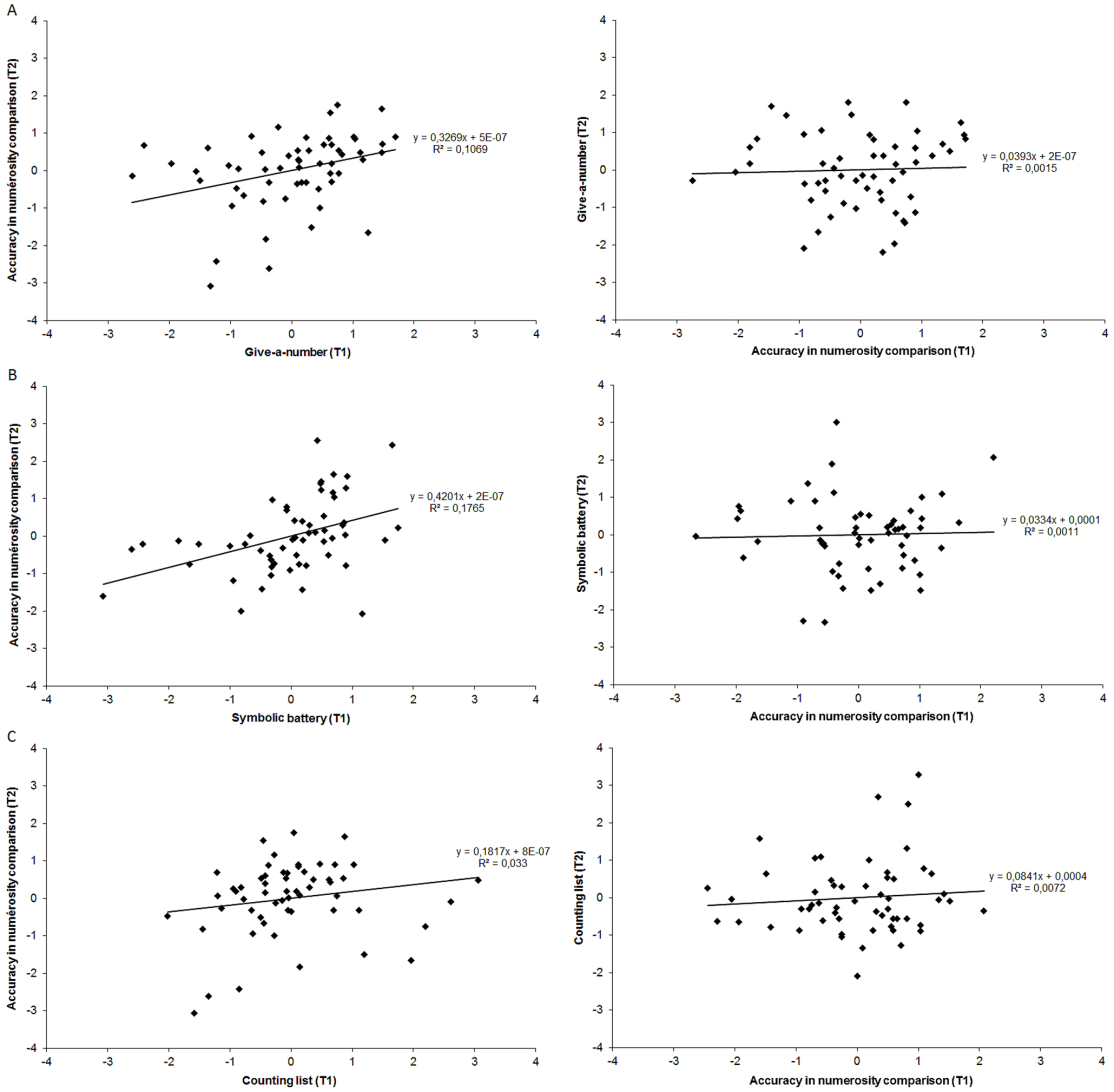
The bidirectional relationships between standardized residuals (controlling for age and the contribution of the autoregressor) for ANS acuity (accuracy in numerosity comparison) and the give-a-number score (Panel A), the symbolic battery score (Panel B), or the counting list (Panel C).

A first series of partial correlation analyses were conducted after controlling for age and the initial score for the predicted variable (thereafter the autoregressor). Indeed, because each pair of measures correlated at time point 1, it is possible that cross-lagged correlations would be partly driven by the simple improvement on the predicted variable with time. Figure 2 depicts the bidirectional relationships between standardized residuals (controlling for age and the contribution of the autoregressor) for accuracy in numerosity comparison and the three symbolic number measures. First consider the cross-lagged correlation between cardinality proficiency (i.e., the give-a-number score) and the accuracy in numerosity comparison. While significant correlations were found between the give-a-number score at time point 1 and accuracy in numerosity comparison at time point 2 (*r*(53)  = .33, *p* = .01), the reverse partial correlation between accuracy in numerosity comparison at time point 1 and the give-a-number score at time point 2 was close to zero (*r*(53)  = .04, *p* = .78). When the two partial correlation coefficients were compared, the difference was highly significant (*t*(53)  = 2.61, *p* = .006). Similar results were observed for the symbolic battery. The partial correlation between the symbolic battery score at time point 1 and accuracy in numerosity comparison at time point 2 was significant (*r*(53)  = .42, *p* = .001) whereas the partial correlation between accuracy in numerosity comparison at time point 1 and the symbolic battery score at time point 2 was not (*r*(53)  = .03, *p* = .81). The two correlation coefficients differed significantly from each other (*t*(53)  = 4.03, *p*<.001). Although the cross-lagged partial correlations between the counting list and accuracy in numerosity comparison showed the same trend (i.e., greater correlation between the counting list at time point 1 and accuracy in numerosity comparison at time point 2, *r*(53)  = .18, *p* = .18, than the reverse, *r*(53)  = .08, *p* = .54) none of them were significant and the two coefficients did not differ from each other (*t*(53)  = 0.75, *p* = .23). Note that the pattern of predictive direction could not be attributed to the impact of the autoregressor as similar trends favoring a greater relationship between score on give-a-number or on symbolic battery at time point 1 and accuracy in numerosity comparison at time point 2 than the reverse were found when partial correlations were controlled for age only.

To distinguish further between the two hypotheses, additional partial correlations were run after controlling for age, non-verbal intelligence, naming performance, visuospatial and verbal spans, as well as the contribution of the autoregressor. The scores on the give-a-number task (*r*(44)  = .27, *p* = .07) and the symbolic battery (*r*(44)  = .37, *p* = .01) at time point 1 were predictively associated with accuracy in numerosity comparison at time point 2, but the respective reverse correlations were close to zero (for the give-a-number task: (*r*(44)  = .0, *p* = .99; for the symbolic battery: (*r*(44)  = .01, *p* = .95). In both cases, the two correlations differed significantly from each other (for the give-a-number task: *t*(44)  = 2.39, *p* = .01; for the symbolic battery: *t*(44)  = 3.60, *p*<.001). Regarding counting skills, children's ability to produce a long correct sequence at time point 1 was not significantly related to high accuracy in numerosity comparison at time point 2 (*r*(44)  = .03, *p* = .85). In the same way, children's performance in numerosity comparison at time point 1 was not associated with their counting list at time point 2 (*r*(44)  = .10, *p* = .50). The two coefficients did not differ from each other (*t*(44)  = −.52, *p* = .30).

#### Symbolic number measures and the Weber fraction

Consider now the Weber fraction as a measure of ANS acuity rather than correct response rates. The partial cross-lagged correlations controlled for age and the contribution of the autoregressor showed a clear pattern of predictive direction between the *w* parameter and the symbolic battery score only. As observed for accuracy in numerosity comparison, while the symbolic scores at time point 1 predicted significantly the *w* parameter at time point 2 even when controlled for age and the initial value of the *w* parameter (*r*(46)  = −.48, *p* = .001), the reverse correlation was not significant (*r*(46)  = −.04, *p* = .78). The two coefficients differed significantly from each other (*t*(46)  = 2.13, *p* = .02). The other partial cross-lagged correlations between the *w* parameter and the give-a-number score or the counting list were not significant, in any direction and did not differ from each other (all, *p*s >.17).

The final set of partial correlations were conducted to examine the relationships between the three symbolic number measures and the Weber fraction after controlling for age, the autoregressor, and general cognitive factors. Once again, the symbolic battery score at time point 1 was predictively related to the *w* parameter at time point 2 (*r*(40)  = −.38, *p* = .01). By contrast, the *w* parameter at time point 1 was not a significant predictor of the symbolic battery score at time point 2 (*r*(40)  = .06, *p*  = .69). The two coefficients differed significantly from each other (*t*(40)  = 2.04, *p* = .02). Beyond the symbolic battery, no predictive relations were found between the *w* parameter and the give-a-number score or the counting list since no correlations were significant in any direction (all, *p*s >.05).

#### Correlations by age group

Table 5 provides simple and partial correlations between the three symbolic measures and the two indexes of ANS acuity across time points, separately for the two age groups. In 4-year-olds, the relationships between the score on the give-a-number task or symbolic battery at time point 1 and accuracy in numerosity comparison at time point 2 were stronger than the reverse relationships irrespectively of whether or not correlations were controlled for the contribution of the autoregressor. It should be yet noted that the difference between the strength of correlations was significant for the give-a-number score, but not for the symbolic battery score. However, taken the argument used by Gathercole and colleagues [38], the fact that only the correlation between symbolic battery at time point 1 and accuracy in numerosity comparison at time point 2 reached significance (but not the reverse correlation) could be taken as an index of predictive direction. The relationships between the score on the give-a-number task or symbolic battery and the *w* parameter followed the same direction, although to a smaller extent for the give-a-number score. When we examined similar correlations in 3-year-olds, no clear predictive direction emerged.

**Table 5:** Bidirectional simple and partial (corrected for the contribution of the autoregressor) correlations between the symbolic measures (give-a-number task, symbolic battery, and counting list) and the indexes of ANS acuity (accuracy and the Weber fraction) across the two time points for each age group.

Age			Simple correlations			Partial correlations		
	*Time point 1*	*Time point 2*	*df*	*r*	*t*	*df*	*r*	*t*
3 years	Give-a-number	Accuracy	16	.43	0.60	13	.38	1.17
	Accuracy	Give-a-number	16	.25		13	.02	
	Battery	Accuracy	16	.13	−0.06	13	.06	0.00
	Accuracy	Battery	16	.15		13	.06	
	Counting	Accuracy	16	.27	0.09	13	.23	0.20
	Accuracy	Counting	16	.24		13	.16	
	Give-a-number	Weber fraction	14	−.15	0.35	7	−.02	0.59
	Weber fraction	Give-a-number	14	−.32		7	−.31	
	Battery	Weber fraction	14	−.04	0.53	7	.26	0.97
	Weber fraction	Battery	14	−.31		7	−.23	
	Counting	Weber fraction	14	.27	0.55	7	.30	0.25
	Weber fraction	Counting	14	.01		7	.18	
4 years	Give-a-number	Accuracy	41	.38**	1.78*	38	.24	2.88**
	Accuracy	Give-a-number	41	.09		38	−.22	
	Battery	Accuracy	41	.38**	0.89	38	.29†	1.40†
	Accuracy	Battery	41	.22		38	.03	
	Counting	Accuracy	41	.14	−0.26	38	.01	−0.36
	Accuracy	Counting	41	.19		38	.08	
	Give-a-number	Weber fraction	40	−.35	−0.12	37	−.23	−0.75
	Weber fraction	Give-a-number	40	−.32		37	−.02	
	Battery	Weber fraction	40	−.50	−1.11	37	−.45	−1.59†
	Weber fraction	Battery	40	−.25		37	−.07	
	Counting	Weber fraction	40	−.30	−0.60	37	−.24	−0.78
	Weber fraction	Counting	40	−.15		37	−.04	

† .05<*p*<.10, *p<.05, **p = .01.

## Discussion

The present study was designed to examine bidirectional relationships between three different symbolic number measures (give-a-number task, symbolic battery, counting list) and ANS acuity in 3-4 years old children. To that aim, we compared the cross-lagged correlations across two time points. This method is widely used in many areas of scientific research in the analysis of longitudinal data [52], [53], especially for identifying reciprocal influences of different cognitive abilities during development [54], [55]. However, some authors have also pointed out the limits of this analysis and the need to interpret the differences between cross-lagged correlations with caution [56]. Therefore, the relationships between symbolic number measures and ANS acuity reported here were discussed in terms of predictive direction rather than causality.

Our main finding is that the impact of cardinality proficiency on the precision of the ANS measured at seven months interval was greater than the reverse influence of the ANS acuity on the later score on the give-a-number task. Similar results were obtained with the symbolic battery. The pattern of predictive direction held when correlations were controlled for the contribution of the autoregressor and general cognitive factors. In that case, the inter-individual variability in the score on the give-a-number task or symbolic battery at time point 1 predicted the differences in numerosity comparison's accuracy at time point 2, while the variability across participants in numerosity comparison's accuracy during the first assessment was not a significant predictor of later performance in symbolic measures.

Although our paper provides evidence of a directional relationship between ANS acuity and symbolic number knowledge, several points need to be clarified. First, we failed to find a clear pattern of predictive direction between children's counting list and ANS acuity. Even within each time point, the ability to count as far as possible showed weak correlations or sometimes no link with accuracy in numerosity comparison. The lack of such relationships reported here and elsewhere in preschoolers [20] could indicate that the number word sequence and the precision of the ANS are not dependent on each other early in the development. Alternatively, it is also possible that the level of knowledge on number sequence at the age of 3-4 is not sufficient to affect ANS acuity. Indeed, some understanding of number word meaning seems necessary to succeed above chance on a numerosity comparison task [19], [20].

An intriguing finding of our study involves the use of the Weber fraction as the traditional index of ANS acuity. The score on the symbolic battery at time point 1 was predictively related to performance in numerosity comparison irrespectively of whether correct response rates or *w* parameters were used. By contrast, cardinality proficiency was not linked to the same extent to these two measures. More precisely, the score on the give-a-number task at time point 1 was related to accuracy in numerosity comparison seven months later but not to the *w* parameter. Mazzocco and her colleagues [33] also reported that accuracy in numerosity comparison but not the *w* parameter was associated with later symbolic number performance in preschoolers. These authors interpreted their failure to observe significant correlations with the *w* parameter as caused by the volatile fits of individual performance by the psychophysical model in young children (a similar conclusion has been proposed in dyscalculic children [57]). It is possible that the number of trials was not sufficient to obtain a reliable measure of the *w* parameter [58]. Alternatively, the different patterns of predictive direction reported here could be due to the fact that the two measures of ANS acuity do not possess the same sensitivities. In particular, the Weber fraction could be a less useful measure of young children's precision than the more classical correct response rates. Indeed, we reported weak correlations between the Weber fraction at time point 1 and Weber fraction at time point 2. In the same vein, recent data indicate that the Weber fraction has also poorer test-retest reliability than a simple accuracy measure even in adults [59].

Another point concerns potential age-related differences across our participants. A close look at the data revealed that the pattern of predictive direction appeared mainly in older children. The score on the give-a-number task or symbolic battery was predictively related to accuracy in numerosity comparison for 4-year-olds. In younger children, a similar trend appeared between the give-a-number score at time point 1 and accuracy in numerosity comparison at time point 2, but the correlation was not significantly stronger than the reverse relationship. The lack of such a pattern of predictive direction between the symbolic battery score and accuracy in numerosity comparison in 3-year-olds could be due to low scores at this age (mean  =  3.6 at time point 1). A plausible interpretation would be that a certain time/level of symbolic knowledge is needed to observe an impact on the performance in numerosity comparison. However, this interpretation must be taken with caution given the small number of participants in this age group.

The present findings shed new insight on the theoretical accounts concerning the association between ANS acuity and symbolic number abilities in young children. According to the dominant theory, the meaning of symbolic numbers is first acquired through recurrent mapping onto the pre-existing magnitude representations [10], [15], [16], [60]. Following this reasoning, one could postulate that the degree of (im)precision of the ANS might influence the acquisition of number words and Arabic digits. In other words, one child who is more accurate in numerosity comparison should develop symbolic number knowledge faster than another child who has a less fine ANS acuity. The rare past studies that used a longitudinal approach were unable to establish a predictive direction between symbolic number abilities and the precision of the ANS because they focused only on one direction. Halberda and his colleagues [24] found that inter-individual differences in ANS acuity at 14 years were retroactively correlated with math scores during childhood. As pointed out by the authors, this result is in line with the dominant view but could also reflect a finer precision of the ANS with progressive engagement in formal maths. In a more recent study, Mazzocco and collaborators [33] reported that the ANS precision measured in 3 to 6 year old children predicted performance in school mathematics two years later and argued that their findings support a directional influence from ANS to symbolic competence. However, the lack of initial measure of children's ability to understand number words or Arabic digits prevents any strong conclusion to be drawn.

The view entertained here is that children who had better knowledge of number words and Arabic digits at an early age were more likely to develop a finer approximate number representation some months later. One crucial question concerns the mechanisms through which the acquisition of exact numbers could influence the ANS. Based on Carey's view [17], [61], [62], symbolic numerals are not constrained by any upper capacity limit and have unrestricted precision, allowing human beings to represent large numbers exactly. A second potential source of refinement comes from the idea that symbolic numbers lead to a more precise access to the ANS than non-symbolic numbers [63]. Following this hypothesis, the overlap between the magnitude representations of adjacent numbers could be reduced through the recurrent mapping with respective symbols. Importantly, our data are not necessarily inconsistent with the dominant theory. The symbolic and non-symbolic number abilities might support and refine each other, in both directions, depending on the age. Because our participants were assessed only at 3–4 years, it is likely that the level of ANS acuity at an earlier stage could explain the variability in the initial skills with symbolic numbers. It is also possible that the symbolic number abilities initially influence the development of the ANS acuity and that, in turn, this refining determines the acquisition of future symbolic number knowledge [33], [35]. What we demonstrated here is that at this age, the comprehension of the exact meaning of number words and Arabic digits is a good index to later precision in numerosity comparison.

Regarding past research on numerical cognition, our hypothesis finds indirect support in several studies on typical and atypical math development. Irrespective of whether or not symbolic numbers are thought to be first acquired by recurrent mapping onto the preverbal number system [15], we can infer from the pattern of cross-lagged correlations reported here that symbolic numbers in turn influence the development of approximate number representation. Therefore, the refinement in numerosity comparison with age [8] could partly reflect contribution from learning of precise number words and Arabic digits through counting and arithmetic. Following this hypothesis, the lack of formal education in math might explain why indigenous adults [9] or illiterate adults whose experience with exact numbers is limited [64] have a weaker ANS acuity than participants who beneficiated from math instruction. Similarly, a poor understanding of symbolic numbers could account for some of the difficulties encountered in developmental dyscalculia. This pervasive learning disability affects both basic numerical competencies [65] and arithmetic skills [66], [67] in 5–7% of school-aged children [68]. Current research indicates that dyscalculic children show weaker performance than typically developing children in symbolic number comparison [69], [70] or in both symbolic and non-symbolic number comparison [57], [71], [72], favouring either a deficit in the access to approximate number representation from symbolic numbers or in this representation *per se*. Importantly, the difficulties in non-symbolic number processing are largely observed in older children [73], suggesting that dyscalculic children are unable to benefit from the increasing precision yielded by symbolic numbers [74]. Regarding rehabilitation program, our data suggest that improving the mapping between symbolic numbers and approximate number representations might help and could even be crucial during the early stages of learning basic numerical knowledge. Further investigation in typically developing school-aged children but also in dyscalculic children is needed to test the potential impact of an intervention on symbolic numbers on the precision of the ANS.
